# Synergy of Extremely Low-Frequency Electromagnetic Fields (ELFEFs) and Sex Hormones Against Oxidative Stress in Multiple Sclerosis

**DOI:** 10.3390/antiox15070851

**Published:** 2026-07-06

**Authors:** Begoña M. Escribano, Manuel E. Valdelvira, Ana Muñoz-Jurado, Montse Feijóo, Eduardo Agüera-Morales, Javier Caballero-Villarraso, Abel Santamaría, Isaac Túnez

**Affiliations:** 1 Department of Cell Biology, Physiology and Immunology, Faculty of Veterinary Medicine, University of Cordoba, 14071 Cordoba, Spain; 2 Maimonides Institute for Research in Biomedicine of Cordoba (IMIBIC), 14004 Cordoba, Spainbc2cavij@uco.es (J.C.-V.);; 3 Department of Biochemistry and Molecular Biology, Faculty of Medicine and Nursing, University of Cordoba, 14071 Cordoba, Spain; mevaldelviradiaz@gmail.com; 4 Department of Medical and Surgery Sciences, Faculty of Medicine and Nursing, University of Cordoba, 14071 Cordoba, Spain; 5 Neurology Service, Reina Sofia University Hospital, 14004 Cordoba, Spain; 6 Analysis Service, Reina Sofia University Hospital, 14004 Cordoba, Spain; 7 Faculty of Sciences, National Autonomous University of Mexico, Mexico City 04510, Mexico; absada@yahoo.com; 8 Department of Healthcare, Metropolitana Autonomous University, Mexico City 04960, Mexico

**Keywords:** experimental autoimmune encephalomyelitis, estrogens, non-invasive brain stimulation techniques, progesterone, testosterone

## Abstract

Transcranial magnetic stimulation (TMS) is a non-invasive brain stimulation method with neuromodulatory capacity in neurodegenerative diseases such as multiple sclerosis (MS). Its therapeutic value is linked to its activity against oxidative stress by activation of antioxidant defenses. The sex hormones, estrogens (E), progesterone (P) and testosterone (T), have demonstrated their power as adjuvants to TMS, improving cortical excitability. The aim of this study was to evaluate the effect of these hormones as adjuvants to extremely low-frequency electromagnetic fields (ELFEFs) in the treatment of experimental autoimmune encephalomyelitis (EAE), the experimental model of MS. The effect of these hormones as replacement therapy was also evaluated in ovariectomized rats treated with ELFEFs. Sixty-five female Dark Agouti rats were divided into 13 groups (5 rats/group), in which biomarkers of oxidative stress and the glutathione redox cycle in non-nervous organs (kidney, liver, heart, intestines and blood) were analyzed. The results show that ELFEFs alone are more effective against oxidative stress. However, P and E were more effective than ELFEFs, both as adjuvants and in hormone replacement therapy, in activating the glutathione system. Therefore, it could be concluded that sex hormones play an important role against MS, enhancing the antioxidant effect of ELFEFs.

## 1. Introduction

Non-invasive brain stimulation (NIBS) methods are innovative tools to investigate the functioning of neural networks and induce short- and long-term changes within them. However, several factors have been identified that affect the variability of the neurophysiological response to brain stimulation techniques, such as age, attention, sex, genetics, and time of day [1,2]. Sex is one of the determining factors of the neuronal, behavioral and cognitive effects induced by these methods [1,3,4]. For example, estrogens have been shown to have a positive impact on cortical excitability in both animals and humans [2,5,6].

Multiple sclerosis (MS) is a chronic, demyelinating, immune-mediated disease of the central nervous system (CNS) that affects 2.8 million people worldwide [7], with a higher prevalence in women. Access of immune cells to the CNS causes an increase in proinflammatory molecules from lymphocytes, inducing the production of reactive oxygen species (ROS) and a depletion of antioxidant defenses [8,9,10]. This causes damage to mitochondria and myelin, oligodendrocyte apoptosis and astrocyte dysfunction, all of which are characteristics of MS [11,12]. Furthermore, in MS, oxidative damage does not only occur in nervous organs. In this regard, Conde et al. (2019) revealed the existence of oxidative stress in non-nervous organs and tissues in experimental autoimmune encephalomyelitis (EAE), an animal model of MS [13].

Different TMS protocols have been developed as a therapeutic tool against neurodegenerative and psychiatric diseases, including MS. There are data showing that those magnetic stimulation protocols, which exert beneficial effects, could trigger an antioxidant action that would favor, at least partially, its therapeutic effect [14].

On the other hand, although women are more susceptible to developing MS, men who develop this disease have greater cognitive impairment and accumulate disability more quickly than women [15]. For this reason, several studies have evaluated the effect of sex hormones in the application of TMS on cortical excitability. The results show that variations in endogenous estrogens, testosterone, and progesterone have modulatory effects on TMS-derived measures of cortical excitability. Specifically, higher levels of estrogens and testosterone have been associated with increased cortical excitability, whereas higher levels of progesterone have been associated with decreased cortical excitability [16]. Rogers and Dhaher (2017) found no effect of sex on the therapeutic utility of rTMS in stroke, where an uninjured hyperexcitable motor cortex can be targeted with slow frequency stimulation (1 Hz rTMS) [17]. However, they only observed differences with respect to sex when visits were grouped by menstrual cycle phase and common hormonal dynamics.

However, the combined effect of sex hormones and TMS on oxidative stress produced by EAE has not been studied in non-nervous tissues and organs, in which the presence of oxidative stress has already been demonstrated during the course of EAE [13]. According to the literature, extremely low-frequency electromagnetic fields (ELFEFs) increased the glutathione antioxidant system [18,19] in EAE rats, against oxidative stress.

Given this lack of evidence, this study has investigated the synergistic use of TMS with sex hormones, with the following objectives: (1) to evaluate estrogens, progesterone and testosterone as adjuvants of ELFEFs in their use as an antioxidant in EAE in non-nervous organs (heart, liver, kidney and intestines) and blood; (2) using estrogens, progesterone, and testosterone together with ELFEFs as replacement therapy in ovariectomized EAE rats to determine antioxidant power in blood, heart, liver, kidney, and intestines; and (3) to verify whether the antioxidant power of hormones and ELFEFs could be derived from the improvement of the antioxidant defenses of the glutathione redox system.

## 2. Materials and Methods

### 2.1. Animals

This study included 65 female Dark Agouti rats supplied by the Animal Experimentation Center (University of Córdoba, Córdoba, Spain). The animals were 12 weeks old, weighing 200–230 g. Animals were housed individually in plastic cages and maintained under controlled conditions (21 ± 2 °C; 12 h light/12 h dark cycle, lights on at 08:00 h), with free access to a standard AIN-93G diet and water throughout the experiment [20]. All experiments were approved by the Bioethics Committee at University of Cordoba (30/03/2017/053) and carried out according to the guidelines of the Directive 86/609/ECC approved by the European Communities Council, the Directive 2010/63/UE of the European Parliament and of the Council and RD 53/2013 passed by the Presidency Minister of Spain (BOE 8 February 2013).

### 2.2. Experimental Groups and Treatments

The rats were divided into 13 experimental groups consisting of five rats. The groups were as follows: control; vehicle; EAE; EAE+Mock; EAE+TMS; EAE+TMS+P (EAE+TMS+progesterone); EAE+TMS+E (EAE+TMS+estrogens); EAE+TMS+T (EAE+TMS+testosterone); EAE+TMS+Sham; EAE+TMS+OVX (EAE+TMS+ ovariectomy); EAE+TMS+OVX+P; EAE+TMS+OVX+E and EAE+TMS+OVX+T.

To induce EAE, animals received a subcutaneous injection (s.c.) at the dorsal base of the tail, consisting of 100 µL of a solution containing 150 µg of myelin oligodendrocyte glycoprotein (MOG; fragment 35–55; Sigma-Aldrich, Madrid, Spain) dissolved in phosphate-buffered saline (PBS) and emulsified (1:1) in complete Freund’s adjuvant (Sigma-Aldrich, St. Louis, Missouri, MO, USA) supplemented with 400 µg of heat-inactivated Mycobacterium tuberculosis (H37Ra, DIFCO, Detroit, MI, USA).

The control group received no treatment. The vehicle group was administered 100 µL of complete Freund’s adjuvant without MOG.

TMS treatment was administered as follows. Animals were placed individually in cylindrical plastic cages designed to minimize movement during exposure. The stimulation system consisted of a pair of Helmholtz coils (Magnetotherapy S.A., Mexico city, Mexico), each composed of 1000 turns of enameled copper wire (7 cm diameter) housed within plastic boxes (10.5 cm × 10.5 cm × 3.5 cm). The coils were placed dorsally and ventrally to the head, with an approximate distance of 6 cm between each coil and the midpoint of the head. Stimulation consisted of a sinusoidal oscillating magnetic field with a frequency of 60 Hz and an amplitude of 0.7 mT ELFEFs. Exposure lasted two hours in the morning, once a day, five days a week (Monday to Friday), for 21 days [18,19,21,22,23]. Animals assigned to the Mock group underwent the same handling and restraint procedures as the stimulated animals but were not exposed to the magnetic field. The aim of having this group was to study the effects of immobilization stress caused by plastic cages [18,19,22,23].

Rats were ovariectomized (OVX), under anesthesia and asepsis conditions using the bilateral procedure described by Poumeau-Delille (1953), or subjected to sham surgery (Sham) [24]. After a 14-day recovery period [25], animals were randomly assigned to the corresponding experimental groups for EAE induction. This design resulted in approximately 28 days between ovariectomy and treatment application, providing a safety margin for the absence of an ovarian cycle, which may persist for up to 20 days [26,27]. Hormonal treatments consisted of 17-β estradiol (2.5 mg/kg of weight (s.c.)) [26], progesterone (8 mg/kg of weight (s.c.)) [27] or testosterone (0.5 mg/kg of body weight (s.c)) [28]. Hormones were administered 5 days a week for 21 days, starting on day 14, coinciding with the application of TMS, in order to assess the role of hormone therapy on the antioxidant capacity of TMS. All reagents were purchased from Sigma (St. Louis, MO, USA).

### 2.3. Sample Preparation

On day 35, animals were sacrificed by decapitation, having previously been anesthetized with an intraperitoneal injection of 75 mg/kg of ketamine (Imalgene^®^ 100 mg/mL, Merial Laboratories, Boehringer Ingelheim S.A, Barcelona, Spain). Blood samples were subsequently collected from the vascular trunk of the neck, using EDTA-K3 tubes. Samples were centrifuged at 3000 rpm for 15 min at 4 °C and the resulting plasma was separated and stored at −85 °C until analysis. Then, under temperature-controlled conditions (4 °C), heart, liver, kidney and intestines were extracted and weighed. Tissue homogenates were immediately prepared with a mechanical homogenizer (Tempest Virtis). All samples were homogenized in Tris (20 mM) at pH 7.4.

### 2.4. Oxidative Stress Parameters

All analyses were performed in duplicate. Oxidative biomarkers in heart, liver, kidney, and intestine were analyzed by spectrophotometry using Bioxytech S.A. reagents (Oxis International; Portland, OR, USA) and a Shimadzu spectrophotometer Shimadzu (UV 1603; Kyoto, Japan). The biomarkers studied included the components of the glutathione redox system: total glutathione (tG; nmol/mg of blood hemoglobin; nmol/mg of protein in the other organs), reduced glutathione (GSH; nmol/mg of blood hemoglobin; nmol/mg of protein in the other organs) and oxidized glutathione (GSSG; nmol/mg of blood hemoglobin; nmol/mg of protein in the other organs). For the determination of glutathione peroxidase (GPx; nmol/mg of hemoglobin in blood; nmol/mg of protein in other organs) the method of Flohé and Gunzler (1984) was used [29]. The biomarkers of oxidative damage studied were lipid peroxidation products (LPO; nmol/mg of blood hemoglobin; nmol/mg of protein in the other organs); carbonylated proteins (CP) which were measured (nmol/g of blood hemoglobin; nmol/g of protein in the other organs) using the method of Levine et al. (1990) and nitric oxide (NOx: total nitrite (nitrite + nitrate); μmol/mg of blood hemoglobin or μmol/mg in the other organs), which was determined using the Griess method [30,31].

Likewise, the GSH/GSSG ratio was determined as a biomarker of antioxidant power.

### 2.5. Statistical Analysis

Data were expressed as mean ± standard deviation (SD). All groups showed a normal distribution, so one-way ANOVA and Bonferroni post hoc were used to determine which specific groups had significant differences (SPSS INC. Version 25 for Windows, Chicago, USA). Comparisons were made between the EAE group and the control group and between the other groups with EAE, EAE+TMS and EAE+TMS+OVX. A Pearson correlation was also performed to verify the relationship between the glutathione redox system and the metabolites produced by oxidative stress. Correlations of LPO and CP with GSH, GSSG, GSH/GSSG ratio and GPx were established. The significance level was *p* < 0.05 for all statistical tests.

## 3. Results

In the evaluation of the results, the vehicle groups are not described, as they show no differences compared to the control group. The results of the EAE+Mock group are also not described, since no differences were observed compared to the EAE group, nor are the results of the EAE+TMS+Sham group described, given that no differences were observed compared to the EAE+TMS group (Supplementary Materials).

### 3.1. Oxidative Stress Parameters (Table 1)

LPO: In all tissues studied there is a significant increase in LPO, in EAE (*p* < 0.001), with respect to control values. When TMS is applied, the EAE+TMS group shows, in all organs, a significant decrease in LPO (*p* < 0.001) when compared to the EAE group. When TMS is combined with hormones, in the EAE+TMS+E, EAE+TMS+P and EAE+TMS+T groups, a reduction in LPO is also seen compared to the EAE group in all tissues. TMS combined with OVX (EAE+TMS+OVX) significantly reduces LPO values compared to EAE (*p* < 0.001) in all organs. However, LPO levels decrease more in the EAE+TMS group (*p* < 0.001) than in EAE+TMS+OVX, except in liver, where there is no significant difference between the EAE+TMS and EAE+TMS+OVX groups. In kidney and heart, the administration of P and E in ovariectomized EAE rats subjected to TMS (EAE+TMS+OVX+P and EAE+TMS+OVX+E) reduces the LPO values with respect to the EAE and EAE+TMS+OVX groups, but without reaching the low values obtained in the EAE+TMS group, compared to which they also show significant differences. In blood and liver, LPO levels decrease compared to EAE, but the decrease is smaller than in the EAE+TMS and EAE+TMS+OVX groups, compared to which they present significant differences. In the intestines, the EAE+TMS+OVX+E and EAE+TMS+OVX+P groups show a decrease in LPO values compared to the EAE and EAE+TMS+OVX groups. Testosterone administration together with TMS treatment in all tissues, for ovariectomized rats (EAE+TMS+OVX+T), causes a decrease in LPO values with respect to the EAE group, but the low levels of LPO obtained with EAE+TMS and EAE+TMS+OVX are not reached, except in the heart.

**Table 1:** Oxidative stress biomarkers: lipid peroxidation products (nmol/mg hemoglobin, blood, or nmol/mg protein), carbonylated proteins (nmol/mg hemoglobin, blood, or nmol/mg protein), and nitric oxide (µmol/mg hemoglobin (blood) or µmol/mg protein) in rats with EAE treated with TMS (extremely low-frequency electromagnetic fields (ELFEFs)) plus hormonal therapy in the following groups: control; EAE; EAE+TMS; EAE+TMS+P EAE+TMS+E; EAE+TMS+T; EAE+TMS+OVX; EAE+TMS+OVX+P; EAE+TMS+OVX+E and EAE+TMS+OVX+T in blood, kidney, liver, heart and intestines.

Oxidative Stress Biomarkers					
**Lipid Peroxidation Products (nmol/mg Hemoglobin (Blood) or nmol/mg Protein)**					
	Blood	Kidney	Liver	Heart	Intestines
Control	0.0642 ± 0.0008	0.7520 ± 0.0042	0.7719 ± 0.0006	0.6247 ± 0.0162	0.6846 ± 0.0001
EAE	1.1510 ± 0.0114 ^a^	2.4525 ± 0.0026 ^a^	3.6202 ± 0.0082 ^a^	1.7747 ± 0.0051 ^a^	1.4196 ± 0.0148 ^a^
EAE+TMS	0.0612 ± 0.0050 ^d^	0.7272 ± 0.0084 ^d^	0.5826 ± 0.0014 ^d^	0.6077 ± 0.0017 ^d^	0.6928 ± 0.0026 ^d^
EAE+TMS+P	0.1100 ± 0.0163 ^d^	0.9000 ± 0.0068 ^d,g^	0.5396 ± 0.0223 ^d,g^	0.7228 ± 0.0043 ^d,g^	0.6992 ± 0.0073 ^d^
EAE+TMS+E	0.1162 ± 0.0138 ^d^	0.7544 ± 0.0102 ^d,g^	0.7882 ± 0.0093 ^d,g^	0.6128 ± 0.0183 ^d^	0.7104 ± 0.0053 ^d^
EAE+TMS+T	0.6680 ± 0.1210 ^d,g^	0.8247 ± 0.0148 ^d,g^	1.8258 ± 0.0223 ^d,g^	1.7724 ± 0.0138 ^d,g^	0.7898 ± 0.0499 ^d,g^
EAE+TMS+OVX	0.4000 ± 0.0879 ^d,g^	1.1094 ± 0.0079 ^d,g^	0.5735 ± 0.0116 ^d^	1.5137 ± 0.0086 ^d,g^	0.8082 ± 0.0027 ^d,g^
EAE+TMS+OVX+P	0.5328 ± 0.0390 ^d,g,j^	0.7642 ± 0.0046 ^d,g,j^	0.6802 ± 0.0173 ^d,g,j^	0.6146 ± 0.0191 ^d,g,j^	0.7163 ± 0.0140 ^d,j^
EAE+TMS+OVX+E	0.5216 ± 0.0210 ^d,g,k^	0.7654 ± 0.0073 ^d,g,j^	0.7613 ± 0.0037 ^d,g,j^	0.6093 ± 0.0172 ^d,g,j^	0.6686 ± 0.0065 ^d,j^
EAE+TMS+OVX+T	0.6694 ± 0.0608 ^d,g,j^	1.4084 ± 0.0044 ^d,g,j^	0.7785 ± 0.0099 ^d,g,j^	0.5947 ± 0.0087 ^d,g,j^	1.0186 ± 0.0154 ^d,g,j^
**Carbonylated Proteins (nmol/g Hemoglobin (Blood) or nmol/g Protein)**					
	Blood	Kidney	Liver	Heart	Intestines
Control	0.0085 ± 0.0000	0.0094 ± 0.0000	0.0074 ± 0.0000	0.0056 ± 0.0001	0.0071 ± 0.0000
EAE	0.0918 ± 0.0011 ^a^	0.0653 ± 0.0017 ^a^	0.1235 ± 0.0016 ^a^	0.3960 ± 0.0074 ^a^	0.1472 ± 0.0003 ^a^
EAE+TMS	0.0060 ± 0.0004 ^d^	0.0093 ± 0.0000 ^d^	0.0074 ± 0.0000 ^d^	0.0069 ± 0.0001 ^d^	0.0070 ± 0.0001 ^d^
EAE+TMS+P	0.0097 ± 0.0003 ^d^	0.0092 ± 0.0000 ^d^	0.0076 ± 0.0001 ^d^	0.0099 ± 0.0001 ^d^	0.0070 ± 0.0000 ^d^
EAE+TMS+E	0.0087 ± 0.0005 ^d^	0.0096 ± 0.0003 ^d^	0.0076 ± 0.0001 ^d^	0.0097 ± 0.0001 ^d^	0.0070 ± 0.0000 ^d^
EAE+TMS+T	0.0040 ± 0.0006 ^d^	0.0121 ± 0.0001 ^d,g^	0.0015 ± 0.0000 ^d,g^	0.0111 ± 0.0001 ^d^	0.0088 ± 0.0010 ^d,g^
EAE+TMS+OVX	0.0057 ± 0.0003 ^d^	0.0102 ± 0.0002 ^d^	0.0037 ± 0.0001 ^d,g^	0.0079 ± 0.0002 ^d^	0.0108 ± 0.0001 ^d,g^
EAE+TMS+OVX+P	0.0031 ± 0.0028 ^d^	0.0072 ± 0.0000 ^d,g^	0.0073 ± 0.0000 ^d,j^	0.0092 ± 0.0008 ^d^	0.0071 ± 0.0001 ^d,j^
EAE+TMS+OVX+E	0.0021 ± 0.0002 ^d^	0.0073 ± 0.0001 ^d,g^	0.0074 ± 0.0001 ^d,j^	0.0098 ± 0.0002 ^d^	0.0071 ± 0.0001 ^d,j^
EAE+TMS+OVX+T	0.0082 ± 0.0002 ^d^	0.0103 ± 0.0000 ^d^	0.0081 ± 0.0001 ^d,j^	0.0090 ± 0.0001 ^d^	0.0071 ± 0.0001 ^d,j^
**Nitric Oxide (µmol/mg Hemoglobin (Blood) or µmol/mg Protein)**					
	Blood	Kidney	Liver	Heart	Intestines
Control	21.7760 ± 0.6024	25.0500 ± 0.2840	21.8080 ± 1.0375	27.5600 ± 1.1400	27.6480 ± 0.1171
EAE	71.1960 ± 0.0586 ^a^	216.0850 ± 0.1673 ^a^	171.2400 ± 3.5379 ^a^	167.2920 ± 2.0343 ^a^	165.8680 ± 0.1968 ^a^
EAE+TMS	22.3800 ± 0.1375 ^d^	19.1250 ± 0.0500 ^d^	23.1760 ± 0.0433 ^d^	26.1880 ± 0.7700 ^d^	26.7480 ± 0.1937 ^d^
EAE+TMS+P	17.6200 ± 0.6081 ^d,g^	44.0200 ± 1.4450 ^d,g^	33.5360 ± 4.6341 ^d,g^	33.3840 ± 4.4807 ^d,h^	27.3160 ± 0.5129 ^d^
EAE+TMS+E	17.7560 ± 0.5213 ^d,g^	43.2700 ± 2.7250 ^d,g^	33.3480 ± 2.7955 ^d,g^	33.8760 ± 3.9459 ^d,g^	27.6200 ± 0.2881 ^d^
EAE+TMS+T	15.6020 ± 2.3188 ^d,g^	69.6000 ± 2.8661 ^d,g^	132.2360 ± 3.4215 ^d,g^	156.4400 ± 0.2811 ^d,g^	74.9720 ± 26.7526 ^d,g^
EAE+TMS+OVX	50.6820 ± 0.4695 ^d,g^	32.7300 ± 1.3825 ^d,g^	27.4600 ± 1.2013 ^d^	25.6000 ± 0.8898 ^d^	69.5400 ± 0.5938 ^d,g^
EAE+TMS+OVX+P	16.3040 ± 0.8125 ^d,g,j^	24.1950 ± 0.5490 ^d,g,j^	34.0240 ± 2.4541 ^d,g,k^	32.7160 ± 3.8807 ^d,k^	27.4560 ± 0.3659 ^d,j^
EAE+TMS+OVX+E	16.2000 ± 0.7759 ^d,g,j^	47.4500 ± 2.2004 ^d,g,j^	39.8550 ± 2.4914 ^d,g,j^	40.2400 ± 3.2987 ^d,h,j^	27.5640 ± 0.1688 ^d,j^
EAE+TMS+OVX+T	18.2280 ± 2.0720 ^d,g,j^	187.1150 ± 1.7407 ^d,g,j^	130.0160 ± 3.3235 ^d,g,j^	144.1760 ± 3.0011 ^d,g,j^	87.6600 ± 0.1822 ^d,g,l^

^a^ *p* < 0.001 vs. control; ^d^ *p* < 0.001 vs. EAE; ^g^ *p* < 0.001 vs. EAE+TMS; ^h^ *p* < 0.01 vs. EAE+TMS; ^j^ *p* < 0.001 vs. EAE+TMS+OVX; ^k^ *p* < 0.01 vs. EAE+TMS+OVX; ^l^ *p* < 0.05 vs. EAE+TMS+OVX. EAE: experimental autoimmune encephalomyelitis; TMS: transcranial magnetic stimulation; OVX: ovariectomized rats; P: progesterone; E: estrogens; T: testosterone.

CP: In blood and heart, EAE increases CP with respect to the control, while all treatments used reduce CP with respect to the EAE group, compared to which they show significant differences. In the remaining tissues, TMS alone (EAE+TMS) or after the addition of E, P and T (EAE+TMS+E, EAE+TMS+P and EAE+TMS+T), reduces CP levels with respect to EAE. However, in kidney and intestine, the addition of T (EAE+TMS+T) does not reach the low values of EAE+TMS. However, in liver, CP levels in EAE+TMS+T are even lower than those of EAE+TMS. Ovariectomy together with TMS (EAE+TMS+OVX) reduces CP values, compared to EAE, in kidney, liver and intestines, although in liver the values are much lower than the values obtained in EAE+TMS, while in intestines they are higher. Addition of P, E and T to ovariectomized rats with TMS treatment (EAE+TMS+OVX+P, EAE+TMS+OVX+E and EAE+TMS+OVX+T) reduces CP levels relative to EAE in liver and intestines. In liver, for these groups of rats, CP levels are higher than in the EAE+TMS+OVX group, while in intestines they are lower than in the EAE+TMS+OVX group. In kidney, in the EAE+TMS+OVX+P, EAE+TMS+OVX+E and EAE+TMS+OVX+T groups, CP is reduced compared to the EAE group. In addition, in kidney, in the groups in which P and E are administered (EAE+TMS+OVX+P and EAE+TMS+OVX+E), levels are lower than those of the EAE+TMS group.

NOx: In all tissues, NOx increases with EAE above control values and treatment with TMS (EAE+TMS) reduces these values. In blood, the addition of P, E and T to TMS (EAE+TMS+P, EAE+TMS+E and EAE+TMS+T) decreased NOx values, compared to the EAE group, showing lower values than those obtained in the groups with TMS (EAE+TMS) as a single treatment. However, in liver, kidney and heart and in intestines in the EAE+TMS+T group, the values are higher than those in the EAE+TMS group. Ovariectomy together with TMS (EAE+TMS+OVX) reduces the NOx values of EAE, although they remain higher than those of TMS alone (EAE+TMS), in blood, kidney and intestines. The addition of T to ovariectomized rats treated with TMS (EAE+TMS+OVX+T), in all tissues, reduces NOx levels with respect to EAE, although NOx values are higher than those obtained in the EAE+TMS and EAE+TMS+OVX groups in all tissues, except in blood, where they are lower. P and E, in all tissues, in ovariectomized rats (EAE+TMS+OVX+P and EAE+TMS+OVX+E) also reduce NOx levels significantly, compared to EAE.

### 3.2. Glutathione System

**Figure 1:**
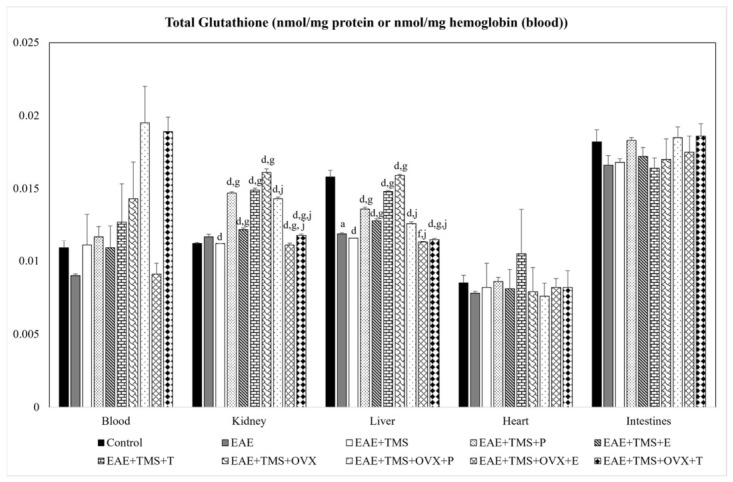
Total glutathione (nmol/mg protein) in EAE rats treated with TMS (extremely low-frequency electromagnetic fields (ELFEFs)) plus hormonal therapy in the following groups: control; EAE; EAE+TMS; EAE+TMS+P; EAE+TMS+E; EAE+TMS+T; EAE+TMS+OVX; EAE+TMS+OVX+P; EAE+TMS+OVX+E and EAE+TMS+OVX+T in blood (nmol/mg hemoglobin), kidney, liver, heart and intestines. ^a^ *p* < 0.001 vs. control; ^d^ *p* < 0.001 vs. EAE; ^f^ *p* < 0.05 vs. EAE; ^g^ *p* < 0.001 vs. EAE+TMS; and ^j^ *p* < 0.001 vs. EAE+TMS+OVX. EAE: experimental autoimmune encephalomyelitis; TMS: transcranial magnetic stimulation; OVX: ovariectomized rats; P: progesterone; E: estrogens; T: testosterone.

tG (Figure 1): Only in the liver there is a significant decrease in tG in the EAE group compared to the control. The application of TMS alone (EAE+TMS) decreases these tG levels compared to EAE. However, TMS combined with hormones (P, E, T; EAE+TMS+P, EAE+TMS+E and EAE+TMS+T) or with ovariectomy (EAE+TMS+OVX) increases tG levels significantly when compared to EAE and EAE+TMS. If ovariectomy is accompanied by hormone therapy (P, E, T; EAE+TMS+OVX+P, EAE+TMS+OVX+E and EAE+TMS+OVX+T), tG levels are significantly reduced compared to EAE+TMS+OVX but remain higher than EAE.

**Figure 2:**
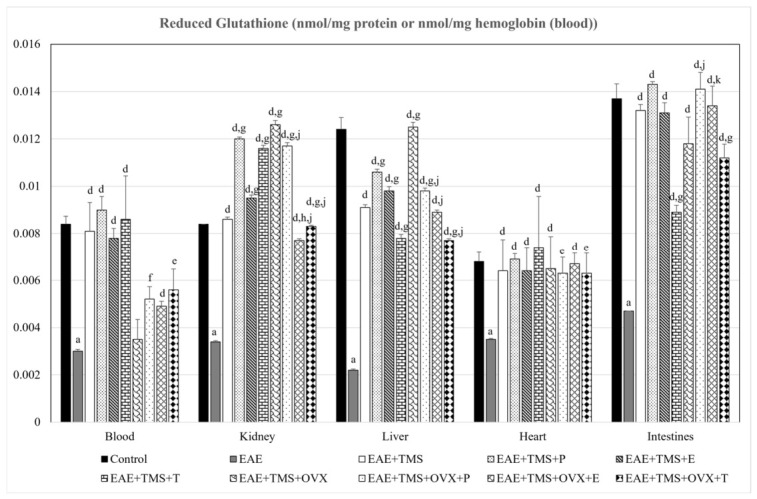
Reduced glutathione (nmol/mg protein) in EAE rats treated with TMS (extremely low-frequency electromagnetic fields (ELFEFs)) plus hormonal therapy in the following groups: control; EAE; EAE+TMS; EAE+TMS+P EAE+TMS+E; EAE+TMS+T; EAE+TMS+OVX; EAE+TMS+OVX+P; EAE+TMS+OVX+E and EAE+TMS+OVX+T in blood (nmol/mg hemoglobin), kidney, liver, heart and intestines. ^a^ *p* < 0.05 vs. control; ^d^ *p* < 0.001 vs. EAE; ^e^ *p* < 0.01 vs. EAE; ^f^ *p* < 0.05 vs. EAE; ^g^ *p* < 0.001 vs. EAE+TMS; ^h^ *p* < 0.01 vs. EAE+TMS; ^j^ *p* < 0.001 vs. EAE+TMS+OVX; and ^k^ *p* < 0.01 vs. EAE+TMS+OVX. EAE: experimental autoimmune encephalomyelitis; TMS: transcranial magnetic stimulation; OVX: ovariectomized rats; P: progesterone; E: estrogens; T: testosterone.

GSH (Figure 2): In all of the tissues studied, EAE reduces GSH levels with respect to the control. In the EAE+TMS group, GSH levels increase significantly with respect to EAE, in all tissues. In liver and kidney, hormone therapy together with TMS (EAE+TMS+P, EAE+TMS+E and EAE+TMS+T), except for T in liver (EAE+TMS+T, which significantly decreases GSH levels compared to EAE+TMS), EAE+TMS+OVX and EAE+TMS+OVX+P, increases GSH levels even more than TMS alone (EAE+TMS). Also, in liver and kidney, in EAE+TMS+OVX+E and EAE+TMS+OVX+T groups, GSH levels are increased compared to EAE, but not as much as in EAE+TMS+OVX, even showing lower levels than in EAE+TMS, except for estrogens, in liver, in which there are no significant differences between EAE+TMS and EAE+OVX+TMS+E groups. In blood and heart, the combination of TMS with hormone therapy (EAE+TMS+P, EAE+TMS+E and EAE+TMS+T) and OVX alone (EAE+TMS+OVX), or with hormone therapy (EAE+TMS+OVX+P, EAE+TMS+OVX+E and EAE+TMS+OVX+T), increase GSH values with respect to EAE (except for OVX in blood -EAE+TMS+OVX-). In intestines, testosterone plus TMS (EAE+TMS+T) or plus TMS and OVX (EAE+TMS+OVX+T), shows lower values than EAE+TMS, but, together with the other therapies, increases GSH values. Also in the latter tissue, EAE+TMS+OVX+P and EAE+TMS+OVX+E show higher GSH levels than EAE+TMS+OVX.

**Figure 3:**
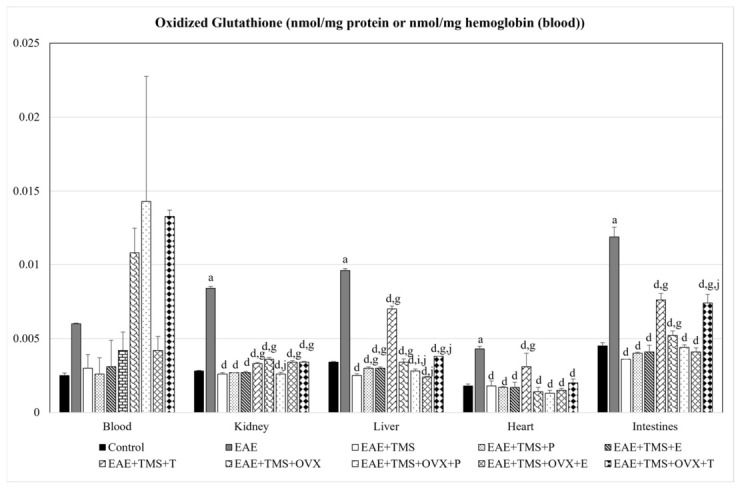
Oxidized glutathione (nmol/mg protein) in EAE rats treated with TMS (extremely low-frequency electromagnetic fields (ELFEFs)) plus hormonal therapy in the following groups: control; EAE; EAE+TMS; EAE+TMS+P EAE+TMS+E; EAE+TMS+T; EAE+TMS+OVX; EAE+TMS+OVX+P; EAE+TMS+OVX+E and EAE+TMS+OVX+T in blood (nmol/mg hemoglobin), kidney, liver, heart and intestines. ^a^ *p* < 0.001 vs. control; ^d^ *p* < 0.001 vs. EAE; ^g^ *p* < 0.001 vs. EAE+TMS; ^i^ *p* < 0.05 vs. EAE+TMS; and ^j^ *p* < 0.001 vs. EAE+TMS+OVX. EAE: experimental autoimmune encephalomyelitis; TMS: transcranial magnetic stimulation; OVX: ovariectomized rats; P: progesterone; E: estrogens; T: testosterone.

GSSG (Figure 3): Except for blood, in which there are no significant differences between groups, in the rest of the organs, EAE increases GSSG levels with respect to the control group. All treatments used, TMS alone (EAE+TMS) and accompanied by OVX (EAE+TMS+OVX) and hormones (EAE+TMS+P, EAE+TMS+E, EAE+TMS+T, EAE+TMS+OVX+P, EAE+TMS+OVX+E and EAE+TMS+OVX+T), reduce GSSG levels with respect to the EAE group. However, T and OVX together with TMS (EAE+TMS+OVX+T) show significantly higher GSSG values than the EAE+TMS group. Also, T together with TMS and OVX (EAE+TMS+OVX+T) shows significantly higher GSSG values than the EAE+TMS+OVX group in liver and intestines.

**Figure 4:**
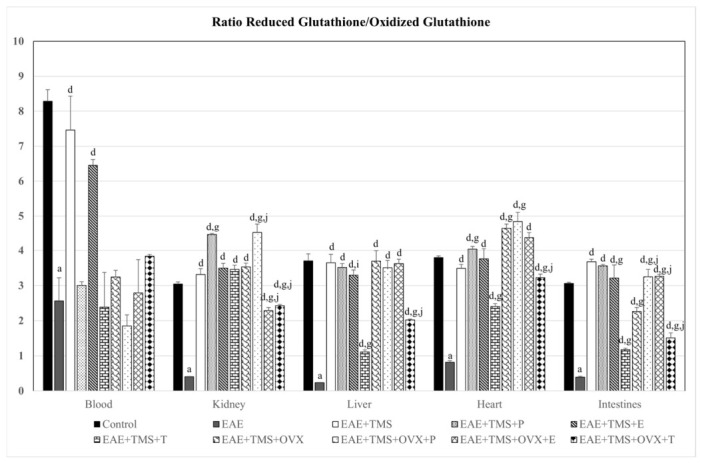
Ratio of reduced glutathione/oxidized glutathione in EAE rats treated with TMS (extremely low-frequency electromagnetic fields (ELFEFs)) plus hormonal therapy in the following groups: control; EAE; EAE+TMS; EAE+TMS+P EAE+TMS+E; EAE+TMS+T; EAE+TMS+OVX; EAE+TMS+OVX+P; EAE+TMS+OVX+E and EAE+TMS+OVX+T in blood, kidney, liver, heart and intestines. ^a^ *p* < 0.001 vs. control; ^d^ *p* < 0.001 vs. EAE; ^g^ *p* < 0.001 vs. EAE+TMS; ^i^ *p* < 0.05 vs EAE+TMS and ^j^ *p* < 0.001 vs. EAE+TMS+OVX. EAE: experimental autoimmune encephalomyelitis; TMS: transcranial magnetic stimulation; OVX: ovariectomized rats; P: progesterone; E: estrogens; T: testosterone.

GSH/GSSG ratio (Figure 4): EAE, in all organs studied, reduces the GSH/GSSG ratio compared to the control group. Except for blood, all treatments, TMS alone (EAE+TMS) or accompanied by hormones (EAE+TMS+P, EAE+TMS+E and EAE+TMS+T) and/or OVX (EAE+TMS+P, EAE+TMS+E, EAE+TMS+T, EAE+TMS+OVX+P, EAE+TMS+OVX+E and EAE+TMS+OVX+T), increase the levels of this ratio with respect to the EAE group. In blood, the only treatments that increase the ratio with respect to the EAE group are TMS plus estrogens (EAE+TMS+E) and TMS alone (EAE+TMS). Testosterone plus TMS (EAE+TMS+T), except in kidney and blood, does not increase the GSH/GSSG ratio values as much as TMS alone (EAE+TMS). Furthermore, in all organs except blood, testosterone plus TMS (EAE+TMS+T) and OVX (EAE+TMS+OVX+T) show lower ratio levels than EAE+TMS+OVX.

**Figure 5:**
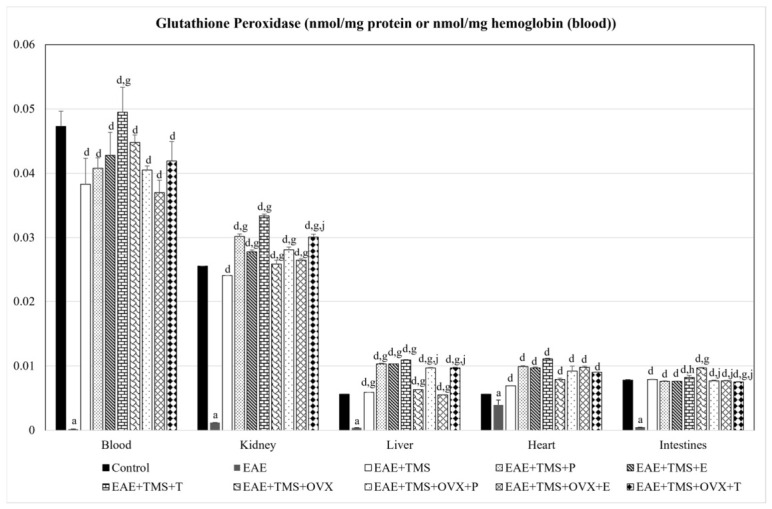
Glutathione peroxidase (nmol/mg protein) in EAE rats treated with TMS (extremely low-frequency electromagnetic fields (ELFEFs)) plus hormonal therapy in the following groups: control; EAE; EAE+TMS; EAE+TMS+P EAE+TMS+E; EAE+TMS+T; EAE+TMS+OVX; EAE+TMS+OVX+P; EAE+TMS+OVX+E and EAE+TMS+OVX+T in blood (nmol/mg hemoglobin), kidney, liver, heart and intestines. ^a^ *p* < 0.001 vs. control; ^d^ *p* < 0.001 vs. EAE; ^g^ *p* < 0.001 vs. EAE+TMS; ^h^ *p* < 0.01 vs. EAE+TMS; and ^j^ *p* < 0.001 vs. EAE+TMS+OVX. EAE: experimental autoimmune encephalomyelitis; TMS: transcranial magnetic stimulation; OVX: ovariectomized rats; P: progesterone; E: estrogens; T: testosterone.

GPx (Figure 5): GPx appears reduced in the EAE group compared to the control group for all organs studied. All treatments, without exception, increase enzyme levels compared to the EAE group.

### 3.3. Pearson Correlations

In all tissues and organs studied, positive correlations (*p* < 0.001) were established between GSSG and LPO, CP and NOx, and negative correlations (*p* < 0.001) were established between GSH, GSH/GSSG ratio and GPx with LPO, CP and NOx.

## 4. Discussion

A recently published review has shown evidence that there are reciprocal interactions between sex hormones, the nervous system and non-invasive brain stimulation techniques. However, this evidence is scarce and was very focused on sex and female hormones, without taking into account male hormones [3]. Furthermore, Veldema (2023) clarifies that most studies are carried out during the menstrual cycle in women, and there is little literature on hormone replacement therapy. Apart from that, all studies are based on cortical excitability tests.

Thus, estrogens have been shown to increase cortical excitability by enhancing glutamate neurotransmission and reducing GABAergic responses [16,32,33]. In contrast, progesterone exhibits an inhibitory action by binding to a steroid-specific site on the GABA-A receptor, facilitating the opening of the chloride channel and elevating the seizure threshold [16,34,35].

Our work allows us to focus on the use of hormone therapy, as well as non-invasive brain stimulation techniques, from another point of view. First, we do not detail the changes in brain connectivity with hormones, but rather we focus on the demonstrated antioxidant power of TMS [18,22] and that of hormonal therapy [26,28]. Likewise, we also focus this evidence on the oxidative stress caused by EAE in non-nervous organs.

It had already been demonstrated by our research group that oxidative stress in EAE rats also occurs in non-nervous organs [13]. In this article, increases in LPO and CP in blood, kidney, liver, heart and intestines are evidenced in EAE rats with respect to the control group. Nitric oxide, which reflects tissue inflammation, is also increased in all of the organs mentioned with the disease. The use of TMS reduces oxidative stress and inflammation in all tissues with respect to EAE, with no evidence that hormonal treatment substantially improves the role of TMS alone. Ovariectomy alone or as hormone replacement therapy (progesterone, estrogens and testosterone), together with TMS, also does not provide different improvements for the reduction of oxidative stress and inflammation, compared to TMS as a sole treatment.

However, the glutathione antioxidant system, in general, does seem to be improved in all tissues studied, except in blood, both with adjuvant therapy and with hormone replacement therapy. In the case of ovariectomized rats administered estrogens and progesterone, there is a greater increase in GSH levels and a greater decrease in GSSG levels than in magnetic stimulation used alone. Testosterone does not appear to be as effective as other hormones as adjuvant therapy for TMS, either in normal or ovariectomized rats, since the increases in GSH and the GSH/GSSG ratio, and the decreases in GSSG, are smaller than with the use of estrogens and progesterone.

Neurodegeneration in MS is a complex process initiated by inflammation, production of ROS and reactive nitrogen species (RNS), and glutamate excitotoxicity leading to demyelination, axon transection and injury, mitochondrial stress, gradual neuron death, and transsynaptic nerve degeneration [15].

TMS was found to decrease lipid peroxidation products and carbonylated proteins and increase the reduced glutathione/oxidized glutathione ratio, which may reflect the redox status of cells. Therefore, it can be established that, at least partially, the effect of TMS application is due to its antioxidant effect [14,18,19,22,36]. These antioxidant effects of TMS could be due to the improvement of antioxidant systems, including glutathione, as demonstrated by the correlations obtained in all organs, which were negative between GPx, GSH and the GSH/GSSG ratio and LPO and CP, and positive between GSSG and LPO and CP.

In addition, as described in other studies, TMS induces an increase in nuclear factor erythroid-2 related factor 2 (Nrf2), which is an important factor in the induction of the body’s antioxidant response and leads to an increase in the expression of antioxidant enzymes. This is one of the possible mechanisms by which TMS could exert its antioxidant effect [14,23,36,37]. Nrf2 is an important transcription factor that recognizes the antioxidant response element to encode key cytoprotective enzymes, such as glutathione peroxidase 1 (GPx1), heme oxygenase 1 (HO-1) and superoxide dismutase 1 (SOD1) [38,39]. It also regulates cytoprotective genes, including the GSH antioxidant pathway, promoting cell survival against cerebral oxidative stress [39,40].

The effect of TMS on Nrf2, mentioned in the previous paragraph as a possible cause of the antioxidant response of the organism, could be enhanced by the co-adjuvant treatment with estrogens and progesterone in our experiment. This has already been demonstrated by our group in a recent study on the role of TMS and the named hormones on the glutathione system in the brain and spinal cord [41].

Regarding hormone therapy, co-treatment of 17β-estradiol (single subcutaneous injection of 10 mg/kg bw) with glutamate (10 mg/kg bw; excess glutamate causes oxidative stress) in 7-day postnatal male Dawley rat pups has been shown to potentially reduce brain oxidative stress by regulating the expression of the Nrf2-mediated cytoprotective enzymes HO-1 and GSH [39]. Furthermore, 17β-estradiol has been reported to increase antioxidant capacity by increasing Nrf2 activity [42,43,44] and by mediating the activities of phase II antioxidant enzymes in the brain [45].

However, adverse effects of long-term estrogen therapy have also been described. Hussen et al. (2024) observed, in 40 adult female BALB/c mice, that 35 days of estrogen therapy (56 μg/kg of body weight per day) caused a significant decrease in the activity of antioxidant markers, including catalase (CAT), GPx and SOD in blood compared to control mice [46]. The authors concluded that estrogen toxicity is the result of prolonged circulation of estrogen molecules in the body, which could be involved in the development of various pathologies in susceptible organs. This fact could not be proven in our animals, since estrogen therapy only lasted 21 days and never acted alone but was administered together with TMS.

On the other hand, few studies describe the role of the other two hormones, progesterone and testosterone, as stimulators of antioxidant systems. Testosterone has been found to block oxidative injury triggered by ovariectomy and ovariectomy together with 3-nitropropionic acid [28], through a receptor-mediated mechanism as reported by Ahlbom et al. (2001). These authors observed that testosterone protects cerebellar granule cells from oxidative stress [47]. Other explanations are due to its aromatization to estradiol [28].

As for progesterone, in the case of MS, the treatment was effective in reducing inflammation and demyelination, leading to improved motor function in a female mouse model [48]. In adult humans, progesterone reduces neuroinflammation, oxidative stress, and brain damage after traumatic brain injury [49].

Taking into account what has been described, we can conclude that: (1) TMS acts as an antioxidant *per se*, through activation of the glutathione antioxidant system, perhaps via Nrf2, in non-nervous organs in EAE rats. (2) The glutathione system appears to improve when TMS is accompanied by hormonal therapy with estrogens, progesterone and testosterone, both as adjuvants and as replacement therapy in ovariectomized rats. (3) Progesterone and estrogens together with TMS, both as adjuvants and in hormone replacement therapy, appear to be more effective than testosterone in activating the glutathione system. (4) The benefits of TMS in MS are due, at least in part, to its effect against oxidative stress.

One hypothesis for future studies could be to study the benefit of TMS therapy used in conjunction with sex hormones in women with MS, both premenopausal and postmenopausal. Further research is also needed to clarify the mechanisms of interaction between TMS and sex hormones and the Nrf2 pathway.

It is worth noting that this study may have several limitations, including: (1) the small number of animals per group could affect the statistical power of the study and increase the risk of overinterpretation of the data; (2) the lack of verification of the molecular pathways through which TMS acts in conjunction with hormone therapy; and (3) in the case of intact females, the estrous cycle was not controlled, which could be relevant to sex hormones and the response to TMS.

## Data Availability

Data will be available in the repository.

## Supplementary Materials

The following supporting information can be downloaded at https://www.mdpi.com/article/10.3390/antiox15070851/s1, (Glutathione Redox System and Oxidative Stress Biomarkers in vehicle; EAE+Mock and EAE+TMS+Sham): Table S1: Mean ± Standard Deviation in Glutathione Redox System: total glutathione (tG; nmol/mg hemoglobin (blood) or nmol/mg protein (other organs)), reduced glutathione (GSH; nmol/mg hemoglobin (blood) or nmol/mg protein (other organs)), oxidized glutathione (GSSG; nmol/mg hemoglobin (blood) or nmol/mg protein (other organs)), glutathione peroxidase (GPx; nmol/mg hemoglobin (blood) or nmol/mg protein (other organs)) and the ratio between GSH/ GSSG in EAE rats in the following groups: vehicle (100 µL of complete Freund’s adjuvant without MOG); EAE+Mock (treated in the same way as those in the TMS group but without receiving real stimulation) and EAE+TMS+Sham (sham-operated) in blood, kidney, liver, heart and intestines. Table S2: Mean ± Standard Deviation in Oxidative Stress Biomarkers: Lipid peroxidation products (LPO; nmol/mg hemoglobin (blood) or nmol/mg protein (other organs)), carbonylated proteins (CP; nmol/g hemoglobin (blood) or nmol/g protein (other organs)) and Nitric Oxide (μmol/mg hemoglobin (blood) or μmol/mg protein (other organs)) in EAE rats in the following groups: vehicle (100 µL of complete Freund’s adjuvant without MOG); EAE+Mock (treated in the same way as those in the TMS group but without receiving real stimulation) and EAE+TMS+Sham (sham-operated) in blood, kidney, liver, heart and intestines.

## Abbreviations

The following abbreviations are used in this manuscript:

TMS	Transcranial magnetic stimulation
MS	Multiple sclerosis
E	Estrogens
P	Progesterone
T	Testosterone
ELFEFs	Extremely low-frequency electromagnetic fields
EAE	Experimental autoimmune encephalomyelitis
NIBS	Non-invasive brain stimulation
rTMS	Repetitive transcranial multiplex stimulation
CNS	Central nervous system
ROS	Reactive oxygen species
OVX	Ovariectomized
MOG	Myelin oligodendrocyte glycoprotein
tG	Total glutathione
GSH	Reduced glutathione
GSSG	Oxidized glutathione
GPx	Glutathione peroxidase
LPO	Lipid peroxidation products
CP	Carbonylated proteins
NOx	Nitric oxide
RNS	Reactive nitrogen species
Nrf2	Nuclear factor erythroid-2 related factor 2
HO-1	Heme oxygenase 1
SOD1	Superoxide dismutase 1

## References

[1] Ridding M.C., Ziemann U. (2010). Determinants of the Induction of Cortical Plasticity by Non-Invasive Brain Stimulation in Healthy Subjects. J. Physiol..

[2] Chung S.W., Thomson C.J., Lee S., Worsley R.N., Rogasch N.C., Kulkarni J., Thomson R.H., Fitzgerald P.B., Segrave R.A. (2019). The Influence of Endogenous Estrogen on High-Frequency Prefrontal Transcranial Magnetic Stimulation. Brain Stimul..

[3] Veldema J. (2023). Non-Invasive Brain Stimulation and Sex/Polypeptide Hormones in Reciprocal Interactions: A Systematic Review. Biomedicines.

[4] Rudroff T., Workman C.D., Fietsam A.C., Kamholz J. (2020). Response Variability in Transcranial Direct Current Stimulation: Why Sex Matters. Front. Psychiatry.

[5] Smith M.J., Adams L.F., Schmidt P.J., Rubinow D.R., Wassermann E.M. (2002). Effects of Ovarian Hormones on Human Cortical Excitability. Ann. Neurol..

[6] Brinton R.D. (2009). Estrogen-Induced Plasticity from Cells to Circuits: Predictions for Cognitive Function. Trends Pharmacol. Sci..

[7] Reich D.S., Lucchinetti C.F., Calabresi P.A. (2018). Multiple Sclerosis. N. Engl. J. Med..

[8] Van Horssen J., Witte M.E., Schreibelt G., de Vries H.E. (2011). Radical Changes in Multiple Sclerosis Pathogenesis. Biochim. Biophys. Acta.

[9] Das U.N. (2012). Is Multiple Sclerosis a Proresolution Deficiency Disorder?. Nutrition.

[10] Sorto-Gomez T.E., Ortiz G.G., Pacheco-Moises F.P., Torres-Sanchez E.D., Ramirez-Ramirez V., Macias-Islas M.A., Celis de la Rosa A., Velázquez-Brizuela I.E. (2016). Effect of Fish Oil on Glutathione Redox System in Multiple Sclerosis. Am. J. Neurodegener. Dis..

[11] Haider L., Fischer M.T., Frischer J.M., Bauer J., Höftberger R., Botond G., Esterbauer H., Binder C.J., Witztum J.L., Lassmann H. (2011). Oxidative Damage in Multiple Sclerosis Lesions. Brain.

[12] Adamczyk-Sowa M., Galiniak S., Zyracka E., Grzesik M., Naparło K., Sowa P., Bartosz G., Sadowska-Bartosz I. (2017). Oxidative Modification of Blood Serum Proteins in Multiple Sclerosis after Interferon Beta and Melatonin Treatment. Oxid. Med. Cell. Longev..

[13] Conde C., Escribano B.M., Luque E., Feijóo M., Caballero-Villarraso J., Valdelvira M.E., Ochoa-Sepúlveda J.J., Lillo R., Paz E., Santamaría A. (2019). Extra-Virgin Olive Oil Modifies the Changes Induced in Non-Nervous Organs and Tissues by Experimental Autoimmune Encephalomyelitis Models. Nutrients.

[14] Medina-Fernández F., Escribano B., Padilla-Del-Campo C., Drucker-Colín R., Pascual-Leone Á., Túnez I. (2018). Transcranial Magnetic Stimulation as an Antioxidant. Free Radic. Res..

[15] Alvarez-Sanchez N., Dunn S.E. (2023). Potential Biological Contributers to the Sex Difference in Multiple Sclerosis Progression. Front. Immunol..

[16] Rivas-Grajales A.M., Barbour T., Camprodon J.A., Kritzer M.D. (2023). The Impact of Sex Hormones on Transcranial Magnetic Stimulation Measures of Cortical Excitability: A Systematic Review and Considerations for Clinical Practice. Harv. Rev. Psychiatry.

[17] Rogers L.M., Dhaher Y.Y. (2017). Female Sex Hormones Modulate the Response to Low-Frequency RTMS in the Human Motor Cortex. Brain Stimul..

[18] Medina-Fernández F., Escribano B., Agüera E., Aguilar-Luque M., Feijoo M., Luque E., Garcia-Maceira F., Pascual-Leone A., Drucker-Colin R., Tunez I. (2017). Effects of Transcranial Magnetic Stimulation on Oxidative Stress in Experimental Autoimmune Encephalomyelitis. Free Radic. Res..

[19] Escribano B., Muñoz-Jurado A., Luque E., Galván A., LaTorre M., Caballero-Villarraso J., Giraldo A.I., Agüera E., Túnez I. (2023). Effect of the Combination of Different Therapies on Oxidative Stress in the Experimental Model of Multiple Sclerosis. Neuroscience.

[20] Reeves P.G., Nielsen F.H., Fahey G.C. (1993). AIN-93 Purified Diets for Laboratory Rodents: Final Report of the American Institute of Nutrition Ad Hoc Writing Committee on the Reformulation of the AIN-76A Rodent Diet. J. Nutr..

[21] Drucker-Colín R., Verdugo-Díaz L., Méndez M., Carrillo-Ruiz J., Morgado-Valle C., Hernández-Cruz A., Corkidi G. (1994). Comparison between Low Frequency Magnetic Field Stimulation and Nerve Growth Factor Treatment of Cultured Chromaffin Cells, on Neurite Growth, Noradrenaline Release, Excitable Properties, and Grafting in Nigrostriatal Lesioned Rats. Mol. Cell. Neurosci..

[22] Medina-Fernández F., Luque E., Aguilar-Luque M., Agüera E., Feijóo M., García-Maceira F., Escribano B., Pascual-Leone Á., Drucker-Colín R., Túnez I. (2017). Transcranial Magnetic Stimulation Modifies Astrocytosis, Cell Density and Lipopolysaccharide Levels in Experimental Autoimmune Encephalomyelitis. Life Sci..

[23] Medina-Fernández F., Escribano B., Luque E., Caballero-Villarraso J., Gomez-Chaparro J., Feijoo M., Garcia-Maceira F., Pascual-Leone A., Drucker-Colin R., Tunez I. (2018). Comparative of Transcranial Magnetic Stimulation and Other Treatments in Experimental Autoimmune Encephalomyelitis. Brain Res. Bull..

[24] Poumeau-Delille G. (1953). Techniques Biologiques en Endocrinologie Expérimentale Chez le Rat.

[25] Teodorov E., Camarini R., Bernardi M.M., Felicio L.F. (2014). Treatment with Steroid Hormones and Morphine Alters General Activity, Sexual Behavior, and Opioid Gene Expression in Female Rats. Life Sci..

[26] Túnez I., Collado J.A., Medina F.J., Peña J., Muñoz M.D.C., Jimena I., Franco F., Rueda I., Feijóo M., Muntané J. (2006). 17 β-Estradiol May Affect Vulnerability of Striatum in a 3-Nitropropionic Acid-Induced Experimental Model of Huntington’s Disease in Ovariectomized Rats. Neurochem. Int..

[27] Ozdamar S., Taskin M.I., Onder G.O., Kaymak E., Baran M., Yay A. (2019). Progesterone Decreases the Extent of Ovarian Damage Caused by Cisplatin in an Experimental Rat Model. Adv. Clin. Exp. Med..

[28] Túnez I., Feijóo M., Collado J.A., Medina F.J., Peña J., Muñoz M.d.C., Jimena I., Franco F., Rueda I., Muntané J. (2007). Effect of Testosterone on Oxidative Stress and Cell Damage Induced by 3-Nitropropionic Acid in Striatum of Ovariectomized Rats. Life Sci..

[29] Flohé L., Günzler W.A. (1984). Assays of Glutathione Peroxidase. Methods Enzymol..

[30] Levine R.L., Garland D., Oliver C.N., Amici A., Climent I., Lenz A.G., Ahn B.W., Shaltiel S., Stadtman E.R. (1990). Determination of Carbonyl Content in Oxidatively Modified Proteins. Methods Enzymol..

[31] Ricart-Jané D., Llobera M., López-Tejero M.D. (2002). Anticoagulants and Other Preanalytical Factors Interfere in Plasma Nitrate/Nitrite Quantification by the Griess Method. Nitric Oxide-Biol. Chem..

[32] François-Bellan A.M., Segu L., Héry M. (1989). Regulation by Estradiol of GABAA and GABAB Binding Sites in the Diencephalon of the Rat: An Autoradiographic Study. Brain Res..

[33] Foy M.R., Xu J., Xie X., Brinton R.D., Thompson R.F., Berger T.W. (1999). 17beta-Estradiol Enhances NMDA Receptor-Mediated EPSPs and Long-Term Potentiation. J. Neurophysiol..

[34] Majewska M.D., Harrison N.L., Schwartz R.D., Barker J.L., Paul S.M. (1986). Steroid Hormone Metabolites Are Barbiturate-like Modulators of the GABA Receptor. Science.

[35] Paul S.M., Purdy R.H. (1992). Neuroactive Steroids. FASEB J..

[36] Zhou X., Li K., Chen S., Zhou W., Li J., Huang Q., Xu T., Gao Z., Wang D., Zhao S. (2022). Clinical Application of Transcranial Magnetic Stimulation in Multiple Sclerosis. Front. Immunol..

[37] Tasset I., Pérez-Herrera A., Medina F.J., Arias-Carrión Ó., Drucker-Colín R., Túnez I. (2013). Extremely Low-Frequency Electromagnetic Fields Activate the Antioxidant Pathway Nrf2 in a Huntington’s Disease-like Rat Model. Brain Stimul..

[38] Itoh K., Chiba T., Takahashi S., Ishii T., Igarashi K., Katoh Y., Oyake T., Hayashi N., Satoh K., Hatayama I. (1997). An Nrf2/Small Maf Heterodimer Mediates the Induction of Phase II Detoxifying Enzyme Genes through Antioxidant Response Elements. Biochem. Biophys. Res. Commun..

[39] Khan I., Saeed K., Jo M.G., Kim M.O. (2021). 17-β Estradiol Rescued Immature Rat Brain against Glutamate-Induced Oxidative Stress and Neurodegeneration via Regulating Nrf2/HO-1 and MAP-Kinase Signaling Pathway. Antioxidants.

[40] Harvey C.J., Thimmulappa R.K., Singh A., Blake D.J., Ling G., Wakabayashi N., Fujii J., Myers A., Biswal S. (2009). Nrf2-Regulated Glutathione Recycling Independent of Biosynthesis Is Critical for Cell Survival during Oxidative Stress. Free Radic. Biol. Med..

[41] Escribano B.M., Valdevira M.E., Muñoz-Jurado A., Feijóo M., Agüera E., Caballero-Villarraso J., LaTorre M., Giraldo A.I., Santamaría A., Túnez I. (2025). The Impact of Sex Hormones on Transcranial Magnetic Stimulation Against the Oxidative Stress in the Pathogenesis of Multiple Sclerosis. Biomolecules.

[42] Wu J., Williams D., Walter G.A., Thompson W.E., Sidell N. (2014). Estrogen Increases Nrf2 Activity through Activation of the PI3K Pathway in MCF-7 Breast Cancer Cells. Exp. Cell Res..

[43] Khan M., Ullah R., Rehman S.U., Shah S.A., Saeed K., Muhammad T., Park H.Y., Jo M.H., Choe K., Rutten B.P.F. (2019). 17β-Estradiol Modulates SIRT1 and Halts Oxidative Stress-Mediated Cognitive Impairment in a Male Aging Mouse Model. Cells.

[44] Song C.H., Kim N., Kim D.H., Lee H.N., Surh Y.J. (2019). 17-β Estradiol Exerts Anti-Inflammatory Effects through Activation of Nrf2 in Mouse Embryonic Fibroblasts. PLoS ONE.

[45] Stakhiv T.M., Mesia-Vela S., Kauffman F.C. (2006). Phase II Antioxidant Enzyme Activities in Brain of Male and Female ACI Rats Treated Chronically with Estradiol. Brain Res..

[46] Hussen T., Al-Shaeli S., Al-Mahna B., Gharban H. (2024). Biochemical and Histological Effects of Long-Term Administration of Estrogen on Female Mice. Adv. Anim. Vet. Sci..

[47] Ahlbom E., Prins G.S., Ceccatelli S. (2001). Testosterone Protects Cerebellar Granule Cells from Oxidative Stress-Induced Cell Death through a Receptor Mediated Mechanism. Brain Res..

[48] Gutzeit O., Segal L., Korin B., Iluz R., Khatib N., Dabbah-Assadi F., Ginsberg Y., Fainaru O., Ross M.G., Weiner Z. (2021). Progesterone Attenuates Brain Inflammatory Response and Inflammation-Induced Increase in Immature Myeloid Cells in a Mouse Model. Inflammation.

[49] MacIntyre D.A., Sykes L., Teoh T.G., Bennett P.R. (2012). Prevention of Preterm Labour via the Modulation of Inflammatory Pathways. J. Matern. Fetal. Neonatal Med..

