# Supplementary material for: Synergy of Extremely Low-Frequency Electromagnetic Fields (ELFEFs) and Sex Hormones Against Oxidative Stress in Multiple Sclerosis
Source: Antioxidants (Basel). 2026 Jul 6;15(7):851. doi: 10.3390/antiox15070851 (PMC13403973; doi:10.3390/antiox15070851)
Supplement: Supplementary file 1 [file antioxidants-15-00851-s001.zip › antioxidants-4347488-supplementary.pdf]

Glutathione Redox System			
**Blood**			
	Vehicle	EAE+Mock	EAE+TMS+Sham
tG (nmol/mg hemoglobin)	0.010±0.001	0.012±0.001	0.011±0.001
GSH (nmol/mg hemoglobin)	0.010±0.001	0.004±0.001	0.008±0.001
GSSG (nmol/mg hemoglobin)	0.001±0.000	0.009±0.001	0.003±0.001
GSH/GSSG	7.607±1.150	1.518±1.217	3.992±0.364
GPx (nmol/mg hemoglobin)	0.045±0.009	0.000±0.000	0.043±0.002
**Kidney**			
	Vehicle	EAE+Mock	EAE+TMS+Sham
tG (nmol/mg protein)	0.011±0.000	0.011±0.000	0.011±0.000
GSH (nmol/mg protein)	0.008±0.000	0.003±0.000	0.008±0.000
GSSG (nmol/mg protein)	0.003±0.000	0.008±0.000	0.002±0.000
GSH/GSSG	3.293±0.092	0.413±0.010	3.493±0.059
GPx (nmol/mg protein)	0.025±0.000	0.001±0.000	0.030±0.001
**Liver**			
tG (nmol/mg protein)	0.013±0.001	0.015±0.001	0.013±0.000
GSH (nmol/mg protein)	0.011±0.000	0.003±0.000	0.010±0.001
GSSG (nmol/mg protein)	0.002±0.000	0.012±0.001	0.003±0.000
GSH/GSSG	4.809±0.058	0.241±0.003	3.486±0.032
GPx (nmol/mg protein)	0.006±0.000	0.000±0.000	0.006±0.000
**Heart**			
tG (nmol/mg protein)	0.008±0.000	0.009±0.001	0.009±0.001
GSH (nmol/mg protein)	0.006±0.000	0.004±0.000	0.007±0.001
GSSG (nmol/mg protein)	0.002±0.000	0.005±0.001	0.002±0.000
GSH/GSSG	3.646±0.0115	0.893±0.029	3.470±0.223
GPx (nmol/mg protein)	0.007±0.000	0.394±0.017	0.008±0.000
**Intestines**			
tG (nmol/mg protein)	0.016±0.000	0.018±0.001	0.018±0.001
GSH (nmol/mg protein)	0.012±0.000	0.005±0.000	0.014±0.001
GSSG (nmol/mg protein)	0.004±0.000	0.013±0.001	0.004±0.000
GSH/GSSG	3.092±0.014	0.361±0.016	3.622±0.271
GPx (nmol/mg protein)	0.008±0.000	0.001±0.000	0.008±0.001

Table S1. Mean ± Standard Deviation in Glutathione Redox System: total glutathione (tG; nmol/mg hemoglobin in blood and nmol/mg protein in the other organs), reduced glutathione (GSH; nmol/mg hemoglobin in blood and nmol/mg protein in the other organs), oxidized glutathione (GSSG; nmol/mg hemoglobin in blood and nmol/mg protein in the other organs), glutathione peroxidase (GPx; nmol/mg hemoglobin in blood and nmol/mg protein in the other organs) and the ratio between GSH/ GSSG in EAE rats in the following groups: vehicle (100 µl of complete Freund's adjuvant without MOG); EAE+Mock (treated in the same way as those in the TMS group but without receiving real stimulation) and EAE+TMS+Sham (sham-operated) in blood, kidney, liver, heart and intestines.

EAE: experimental autoimmune encephalomyelitis; TMS: transcranial magnetic stimulation (extremaly low-frecuency electromagnetic fields (ELFEFs)); MOG: myelin oligodendrocyte glycoprotein

Oxidative Stress Biomarkers			
**Blood**			
	Vehicle	EAE+Mock	EAE+TMS+Sham
LPO (nmol/mg hemoglobin)	0.060±0.003	1.102±0.052	0.066±0.004
CP (nmol/g hemoglobin)	0.008±0.002	0.104±0.010	0.009±0.001
Nitric Oxide (µmol/mg hemoglobin)	22.836±0.575	72.800±1.369	21.934±0.871
**Kidney**			
LPO (nmol/mg protein)	0.739±0.011	2.456±0.006	0.733±0.001
CP (nmol/g protein)	0.009±0.000	0.065±0.002	0.009±0.000
Nitric Oxide (µmol/mg protein)	23.700±0.300	213.155±0.0397	23.730±0.805
**Liver**			
LPO (nmol/mg protein)	0.757±0.004	3.806±0.021	0.609±0.015
CP (nmol/g protein)	0.007±0.000	0.125±0.001	0.008±0.000
Nitric Oxide (µmol/mg protein)	21.832±0.763	173.552±1.281	27.044±2.141
**Heart**			
LPO (nmol/mg protein)	0.625±0.011	1.771±0.005	0.555±0.011
CP (nmol/g protein)	0.007±0.000	0.394±0.017	0.008±0.000
Nitric Oxide (µmol/mg protein)	27.420±0.227	166.092±1.510	28.904±1.232
**Intestines**			
LPO (nmol/mg protein)	0.687±0.002	1.428±0.007	0.684±0.001
CP (nmol/g protein)	0.007±0.000	0.148±0.001	0.007±0.000
Nitric Oxide (µmol/mg protein)	25.380±0.275	165.568±0.452	27.028±0.171

Table S2. Mean ± Standard Deviation in Oxidative Stress Biomarkers: Lipid peroxidation products (LPO; nmol/mg hemoglobin in blood an nmol/mg protein in the other organs), carbonylated proteins (CP; nmol/g hemoglobin in blood and nmol/g protein in the other organs) and Nitric Oxide (µmol/mg hemoglobin in blood and µmol/mg protein in the other organs) in EAE rats in the following groups: vehicle (100 µl of complete Freund's adjuvant without MOG); EAE+Mock (treated in the same way as those in the TMS group but without receiving real stimulation) and EAE+TMS+Sham (sham-operated) in blood, kidney, liver, heart and intestines.

EAE: experimental autoimmune encephalomyelitis; TMS: transcranial magnetic stimulation (extremaly low-frecuency electromagnetic fields (ELFEFs)); MOG: myelin oligodendrocyte glycoprotein
